# Connexin 43 Gene Ablation Does Not Alter Human Pluripotent Stem Cell Germ Lineage Specification

**DOI:** 10.3390/biom12010015

**Published:** 2021-12-22

**Authors:** Grace A. Christopher, Rebecca J. Noort, Jessica L. Esseltine

**Affiliations:** Division of BioMedical Sciences, Faculty of Medicine, Memorial University of Newfoundland, St. John’s, NL A1B 3V6, Canada; gac578@mun.ca (G.A.C.); rjfrohlich@mun.ca (R.J.N.)

**Keywords:** human pluripotent stem cells, connexin channels, germ lineage specification, differentiation, CRISPR-Cas9 gene ablation

## Abstract

During embryonic germ layer development, cells communicate with each other and their environment to ensure proper lineage specification and tissue development. Connexin (Cx) proteins facilitate direct cell–cell communication through gap junction channels. While previous reports suggest that gap junctional intercellular communication may contribute to germ layer formation, there have been limited comprehensive expression analyses or genetic ablation studies on Cxs during human pluripotent stem cell (PSC) germ lineage specification. We screened the mRNA profile and protein expression patterns of select human Cx isoforms in undifferentiated human induced pluripotent stem cells (iPSCs), and after directed differentiation into the three embryonic germ lineages: ectoderm, definitive endoderm, and mesoderm. Transcript analyses by qPCR revealed upregulation of Cx45 and Cx62 in iPSC-derived ectoderm; Cx45 in mesoderm; and Cx30.3, Cx31, Cx32, Cx36, Cx37, and Cx40 in endoderm relative to control human iPSCs. Generated Cx43 (*GJA1*) CRISPR-Cas9 knockout iPSCs successfully differentiated into cells of all three germ layers, suggesting that Cx43 is dispensable during directed iPSC lineage specification. Furthermore, qPCR screening of select Cx transcripts in our *GJA1-/-* iPSCs showed no significant Cx upregulation in response to the loss of Cx43 protein. Future studies will reveal possible compensation by additional Cxs, suggesting targets for future CRISPR-Cas9 ablation studies in human iPSC lineage specification.

## 1. Introduction

The connexin (Cx) family of gap junction-forming proteins facilitates the direct exchange of small messenger molecules between adjacent cells. There are 21 distinct connexin proteins expressed in humans and 20 in mice [1]. Cx proteins form hexameric hemichannels at the cell surface which dock to those on neighboring cells and facilitate gap junction intercellular communication (GJIC) [2,3]. Homomeric and homotypic channels arise from the assembly of a single connexin isoform, while heteromeric and heterotypic channels involve the intermixing of related connexin isoforms within a single hemichannel or gap junction channel. Thus, each channel’s gating and specificity are determined by its subunit composition and Cx isoform topology differences [4,5,6]. Differential Cx expression in discrete cell and tissue types enables precise regulation of cell-to-cell communication across various organs. Moreover, multiple Cxs can be expressed in the same cell types, increasing communication complexity [7]. This high level of Cx isoform intermixing creates a complex hierarchy of channel selectivity to exquisitely regulate cell signaling.

The importance of GJIC in human development is exemplified by the numerous human germline Cx mutations that result in developmental disorders [8]. Cx43 is the earliest expressed connexin in development, appearing at the 2–4 cell embryo stages and persists in many adult tissues [9]. In addition to being the first Cx isoform expressed, Cx43 is by far the best understood isoform in the early embryo. For example, in the pre-implantation embryo, Cx43 channel interaction with cadherins is believed to facilitate the cell–cell contact required for embryo compaction [10,11], although the necessity of Cx43 for compaction is contested [12,13]. Meanwhile, much less is understood about other Cxs in the early embryo and during early lineage specification. Human preimplantation embryos have been reported to express transcripts and proteins for Cx26, Cx31, Cx43, and Cx45 [13,14,15]. The loss of Cx26 is embryonic lethal in mice due to defective transplacental glucose uptake [16]. Meanwhile, loss of Cx43 or Cx45 in mice results in perinatal or embryonic lethality due to cardiac malformations [17,18]. Human preimplantation embryos maintain the expression of Cx32 and Cx43 throughout embryonic development. Meanwhile, Cx26 and Cx45 expression fluctuates during the developmental timeline [19].

Like cells of the early embryo, human and murine stem cells in vitro exhibit connexin expression and GJIC [20]. Mouse and human embryonic stem cells (ESCs), as well as induced pluripotent stem cells (iPSCs), are coupled by Cx43, and Cx45 gap junctions [1,12]. Human ESCs also express transcripts for eighteen out of the twenty-one identified connexin isoforms [13,21]. Inhibiting GJIC significantly decreases iPSC reprogramming efficiency, while pharmacological GJIC inhibition leads to human iPSC cell death [20,22]. While a variety of Cxs are expressed in pluripotent stem cells, it is unclear how these Cx channels affect cell fate decisions and subsequent tissue development.

One of the first cell fate specification events occurs during gastrulation with the emergence of the three embryonic germ layers: ectoderm, mesoderm, and definitive endoderm. Ectoderm gives rise to the central nervous system and epidermis, while mesoderm forms musculature and connective tissues, and definitive endoderm is responsible for producing internal organs, including the pancreas and intestines [23,24]. Dissecting how GJIC contributes to the differentiation of downstream cell types has been challenging. For instance, human ESC differentiation towards hepatocytes relies on Cx32 expression but is further promoted by Cx43 downregulation [25,26]. Cx43 is reportedly upregulated during human ESC differentiation toward endoderm, and continued Cx43 activity promotes differentiation to endoderm-derived pancreatic precursors [27]. However, this reported role of Cx43 in endoderm differentiation is contrasted by the work of Peng and colleagues, wherein Cx43 knockdown impaired mesoderm formation while endoderm formation remained unaltered [22]. Therefore, questions remain as to how connexin-mediated intercellular communication influences lineage specification.

Here, we investigate connexin expression and distribution throughout human pluripotent stem cell differentiation to the three germ layers, focusing on the role of the prototypical Cx43. Our transcript analysis identified 11 of the 21 Cx isoforms in iPSCs and differential expression between the three germ layers. Transcripts for Cx45 and Cx62 were enriched in ectoderm, and Cx45 was elevated in mesoderm, while endoderm displayed increased levels of Cx30.3, Cx31, Cx32, Cx36, Cx37, and Cx40 transcripts relative to iPSCs. Western blotting revealed Cx43 protein expression in iPSCs and the three germ layers with heightened Cx43 expression in mesoderm. Cx43 knockout (*GJA1-/-*) iPSCs, generated using CRISPR-Cas9 gene ablation, maintained characteristics typical of iPSCs and successfully differentiated into cells of ectoderm, mesoderm, or endoderm lineages. Scrape loading dye transfer assays with Lucifer yellow and neurobiotin confirmed depleted GJIC across *GJA1-/-* iPSCs, ectoderm, mesoderm, and endoderm compared to control cells. Therefore, we conclude that Cx43-mediated GJIC is not essential during human iPSC lineage specification.

## 2. Materials and Methods

### 2.1. Induced Pluripotent Stem Cell Culture

These studies were approved by Newfoundland and Labrador Human Research Ethics Board #2018.210. Human female control and *GJA1-/-* iPSCs were obtained through a material transfer agreement with The University of Western Ontario (London, ON, Canada) [20,28,29]. Human male iPSCs were purchased from the Coriell Institute (Cat# GM25256, Camden, NJ, USA). Mono-allelic *EGFP* insertion behind the endogenous *GJA1* allele of GM25256 iPSCs (Cx43-eGFP) was performed by The Allen Institute for Cell Science (Cat# AICS-0053 cl.16, Seattle, WA, USA).

Human iPSCs were housed in a humidified 37°C cell culture incubator supplemented with 5% CO_2_ and fed daily with either Essential 8 media (Cat# A1517001, ThermoFisher, Waltham, MA, USA) or mTeSR Plus (Cat# 100-0276, STEMCELL Technologies, Vancouver, BC, Canada). Pluripotent stem cells were passaged as small aggregates using Gibco™ enzyme-free cell dissociation buffer (Cat# 13151014, ThermoFisher). Cultureware were pre-coated for one hour by diluting cold Geltrex (1:100) (Cat# A1413302, ThermoFisher), according to manufacturer’s instructions in cold DMEM (Cat# 319-015-CL, Wisent, St. Bruno, QC, Canada).

For experiments requiring passage of single cells, iPSCs were incubated at 37 °C in StemPro Accutase (Cat# A1110501, ThermoFisher). Single cells were re-suspended in media containing 10 µM of Y-27632 (Cat#10005583, Cayman Chemicals, Ann Arbor, MI, USA) to promote single-cell survival [30].

### 2.2. Stem Cell Differentiation

Directed iPSC differentiation toward definitive endoderm, mesoderm, or ectoderm was performed using the STEMdiff™ Trilineage Differentiation Kit (Cat# 05230, STEMCELL Technologies), according to the manufacturer’s instructions.

For spontaneous monolayer differentiation, iPSCs were seeded into Geltrex-coated cell culture vessels containing Essential 6 media (Cat# A1516401, ThermoFisher), which lacks the crucial pluripotency growth factors TGF-β and FGF2 [31]. Media were changed every 1–2 days during the spontaneous differentiation process [32], and differentiation was allowed to progress until day nine before processing.

For spontaneous differentiation via embryoid bodies (EBs), 9000 Accutase-digested single-celled iPSCs in Essential 6 fortified with 10 µM of Y-27632 were plated into a 96-well round-bottom plate coated with 1% agarose prepared in deionized water to confer a non-adherent surface [33,34]. EBs were fed with Essential 6 every other day to promote spontaneous differentiation.

### 2.3. Immunofluorescence

Cells were fixed at room temperature for 30 min, with 10% normal buffered formalin (Cat# CA71007-344, VWR, Radnor, PA, USA). Fixed cultures were incubated in primary and secondary antibodies (Table 1) diluted in blocking/permeabilization solution (3% BSA (Cat# 800-095-EL, Wisent) and 0.1% Triton™ X-100 (Cat# T8532, Sigma-Aldrich, St. Louis, MO, USA)). Cells were mounted with #1.5 cover glass with either Prolong™ Diamond Antifade Mountant (Cat# P36962, Life Technologies, Carlsbad, CA, USA), containing DAPI nuclear stain or prepared Mowiol 4-88 (Cat# 81381, Sigma-Aldrich) mounting media containing DABCO antifade compound (Cat# D013425G, Fisher Scientific, Ottawa, ON, Canada), as described by Cold Spring Harbour [35].

### 2.4. Confocal Microscopy

Confocal immunofluorescence images were acquired on an Olympus Fluoview FV10i-W3 confocal microscope (Olympus, Tokyo, Japan) running Fluoview v2.1.1.7 software, equipped with 60X/1.2 NA and 10X/0.4 NA water immersion lenses, and the following lasers to visualize fluorophores: DAPI/Hoechst 33342 (405-nm laser); Alexa Fluor 488/eGFP (473-nm laser); Phalloidin/Alexa Fluor 555 (559-nm laser); and Alexa Fluor 647 (635-nm laser). Laser power and sensitivity were adjusted to visualize immunofluorescence and minimize background signal. Additional sample imaging was performed on an Olympus Fluoview FV1000 confocal microscope fitted with 10X/0.4 NA, 20X/0.75 NA or 40X/0.95 NA lenses and the following lasers: 405 nm, 458 nm, 568 nm, and 633 nm. Images were analyzed using Fiji open-source software. Images were analyzed, pseudo-colored, and made into composites with Fiji software [36]. Brightness and contrast were equally adjusted for optimal visualization in all images using Fiji software. Quantitative analysis of protein colocalization was performed with the JACoP plugin for Fiji which reports Manders’ coefficient values, as described in Bolte et al. (2006) [37].

### 2.5. Western Blotting

Cells were lysed with ice-cold lysis buffer (50 mM Tris-HCl (Cat# M-26956, Fisher Scientific) pH 8, 150 mM of NaCl, 0.02% NaN_3_ (Cat# S2002, Sigma-Aldrich), 0.1% Triton X-100 (Cat# T8532, Sigma-Aldrich), protease inhibitors (2 µg/mL leupeptin (Cat# AAJ6188MB, Fisher Scientific) and 2 µg/mL aprotinin (Cat# AAJ11388MB, Fisher Scientific)), and the phosphatase inhibitors (10 mM NaF (Cat# S299-100, Fisher Chemical) and 1 mM Na_3_VO_4_ (Cat# 81104, AlfaAesar, Haverhill, MA, USA)). Soluble proteins were separated via SDS-PAGE and transferred to a 0.45-µm pore nitrocellulose membrane (Cat# 1620115, BioRad, Hercules, CA, USA). Primary and secondary antibodies were diluted in TBS-T (15.23 mM Tris-HCl, 4.62 mM Tris-Base, 150 mM NaCl, 0.1% Tween-20 adjusted to pH 7.6) with 3% nonfat milk (Table 2). Proteins were visualized with Clarity Western ECL Substrate (Cat# 1705061, BioRad) and imaged on a GE ImageQuant LAS 4000 (28 9558 10, GE Healthcare, Chicago, IL, USA). Quantitative analysis of resolved Western blots was conducted using Fiji software. Protein expression was normalized to the housekeeping gene GAPDH to account for protein loading across samples.

### 2.6. RNA Transcript Analysis

RNA extraction was conducted using the PureLink™ RNA Mini Kit (Cat# 12183025, Thermofisher). Five hundred nanograms of extracted RNA was converted to complementary DNA (cDNA) using the High-Capacity cDNA Reverse Transcription Kit (Cat# 4368814, Thermofisher). Quantitative reverse transcription-polymerase chain reaction (qPCR) analysis was performed using intercalating dye technology (ssoAdvanced SYBR green supermix) (Cat# 1725274, BioRad), according to the manufacturer’s instructions and an annealing temperature of 60 °C. Primer sets were purchased from Integrated DNA Technologies (Newark, NJ, USA) where catalog information and sequences are listed in Table 3. PCR reactions were run on a ViiA™ 7 (ThermoFisher) running QuantStudio Real-Time PCR software version 1.3.

### 2.7. Dye Transfer Assay

Cell monolayers were scratched with a scalpel and incubated in dye-containing media (Hoechst 33342 (1:1000) and 0.5 mg/mL of rhodamine B (Cat# D1841, Fisher Scientific), along with either 1 mg/mL of Lucifer yellow (Cat# L453, Fisher Scientific) or 2 mg/mL neurobiotin (Cat# VECTSP112020, Cedarlane, Burlington, ON, Canada)) for ten minutes in the dark prior to fixation. Neurobiotin was visualized using DyLight streptavidin-488 (1:50) (Cat# 405218, Cedarlane).

Fluorescent images of dye transfer in iPSCs, ectoderm, mesoderm, or endoderm were taken using a Zeiss AxioObserver microscope equipped with an X-Cite Series 120Q lamp (Excelitas Technologies, Mississauga, ON, Canada). Image acquisition used the 5X/0.12 NA A-Plan and 10X/0.25 NA Ph1 objectives. Pseudo-coloring, scale calibration, and analysis of dye transfer was performed with Fiji software [36]. Four or more images were taken per sample, and ten dye migration measurements made per image. Brightness and contrast were equally adjusted for optimal visualization in all images using Fiji software.

### 2.8. Flow Cytometry

Flow cytometry of singularized cells from embryoid bodies was performed on a CytoFLEX (Beckman Coulter, Brea, CA, USA) flow cytometer. Antibodies were titrated over a range of concentrations prior to use and the following controls were included in all flow cytometry assays: unstained control, fluorescence-minus-one (FMO) controls, and single-color compensation controls for fluorochromes. UltraComp compensation beads (Cat# 01-2222-43, ThermoFisher) were used with antibodies raised in mice.

Live single-cell suspensions were labelled with Zombie NIR™ fixable viability dye (Cat# 423105, BioLegend^®^, San Diego, CA, USA) diluted at 1:1000 to eliminate dead cells during the analysis stage. Next, the cells were fixed in 10% normal buffered formalin for 10 minutes at 4 °C followed by permeabilization for 15 minutes at room temperature with Ca^2+^and Mg^2+^-free PBS supplemented with 0.5% BSA and 0.1% Triton X-100. Primary antibodies (used at dilutions according to Table 4) were incubated for 30 min at 4 °C in the dark. Flow cytometric analysis was performed using FlowJo software (version 10.7.1).

### 2.9. Statistical Analysis

Statistical analysis and plotting of raw data were performed using Graph Pad Prism v.8. Graphs presented as ± standard error of the mean (SEM). Unless otherwise stated, n ≥ 3 independent biological replicates. Student’s t-test was performed for statistical analysis between two groups. Larger data sets of three or more groups were analyzed using analysis of variance (ANOVA) with Tukey’s multiple comparisons test where * *p* < 0.05, ** *p* < 0.01, and *** *p* < 0.001.

## 3. Results

### 3.1. Connexin Isoforms Are Differentially Expressed during Human iPSC Germ Lineage Specification

**Figure 1:**
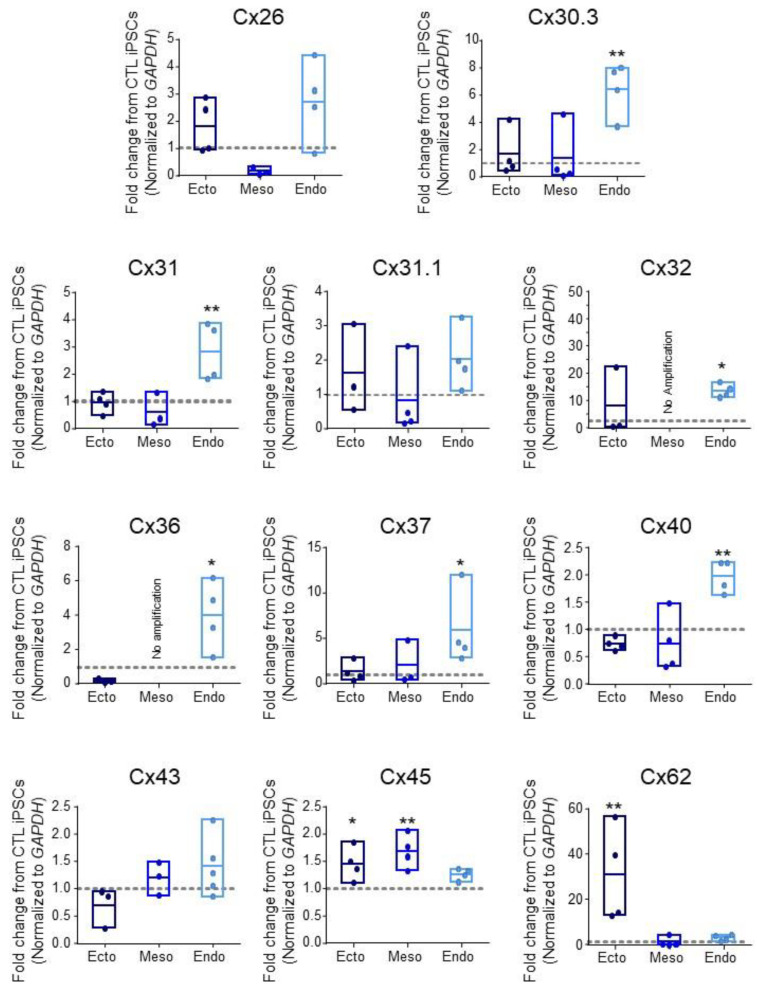
Connexin mRNA expression during human iPSC lineage specification. Quantitative RT-PCR (qPCR) expression of mRNA transcripts encoding 11 of the 21 human connexin isoforms in human control (CTL) iPSCs (grey dotted line) and after directed differentiation into ectoderm, endoderm, and mesoderm. Data represent the standard error of the mean of 3–5 independent experiments. * *p* < 0.05; ** *p* < 0.01 compared to undifferentiated iPSCs.

Given that aberrant GJIC is linked to numerous diseases [8], we investigated the expression of 11 connexin isoforms in human control iPSCs and in their subsequent directed differentiation to the three embryonic germ layers: ectoderm, mesoderm, and definitive endoderm (Figure 1 and Table 5). These isoforms were selected based on previous identification in human PSCs (pluripotent stem cells) or during early lineage restriction [7,21,38]. We differentiated control iPSCs to the three germ layers using the STEMdiff™ Trilineage Differentiation Kit (STEMCELL Technologies) and compared resulting Cx transcript expression patterns. Transcripts encoding Cx26, Cx31.1, and Cx43 showed no significant fluctuation across iPSCs, ectoderm, mesoderm, or endoderm (Figure 1). Ectoderm differentiation was accompanied by a 1.451 ± 0.3001 fold increase in Cx45 transcripts (*p* < 0.05) and 30.68 ± 20.94 fold increase in Cx62 (*p* < 0.01) transcripts compared to undifferentiated iPSCs (Figure 1). Cx45 was also significantly increased in mesoderm at 1.681 ± 0.313 fold over iPSCs (*p* < 0.01), while Cx32 and Cx36 transcripts were present in undifferentiated iPSCs but decreased to become undetectable after iPSC differentiation to mesoderm (Figure 1). Interestingly, endoderm differentiation was accompanied by the upregulation of several connexin species: Cx30.3 increased 6.439 ± 1.963 fold (*p* < 0.01) in endoderm cells compared to undifferentiated iPSCs, while Cx31 mRNA expression increased 2.819 ± 1.063 fold (*p* < 0.01), Cx32 was up 13.63 ± 2.454 fold (*p* < 0.05), Cx36 increased 3.964 ± 2.016 fold (*p* < 0.05), Cx37 was upregulated 5.826 ± 4.144 fold (*p* < 0.05), and Cx40 transcript was elevated 1.974 ± 0.296 fold (*p* < 0.01) (Figure 1 and Table 5). Together, these qPCR screens reveal the exquisite control of connexin isoform expression as cells are assigned to the three embryonic germ lineages.

### 3.2. Cx43 Protein Expression Persists throughout Lineage Specification

**Figure 2:**
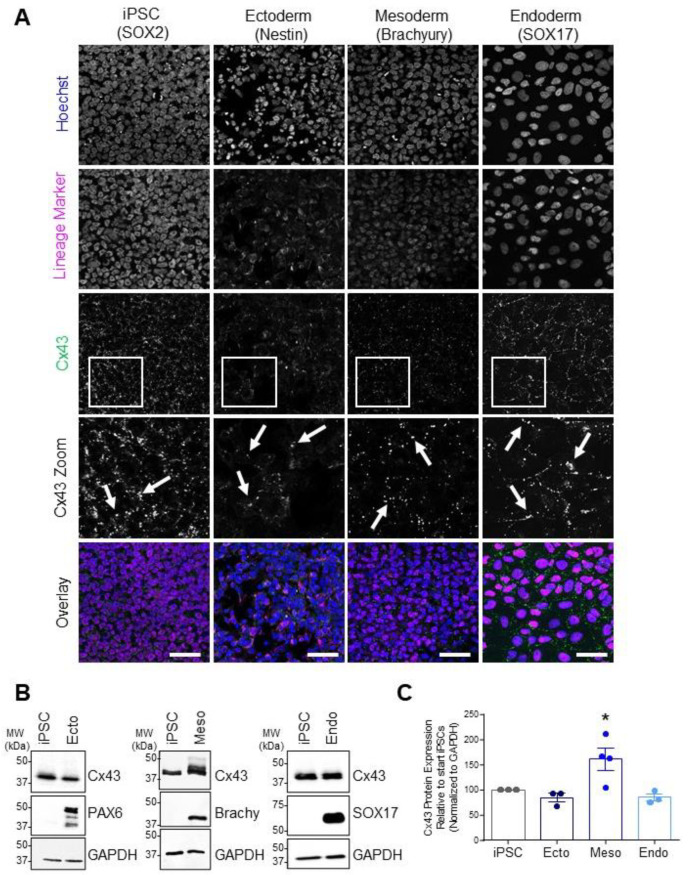
Cx43 protein expression and localization in iPSCs and cells of each germ lineage. (**A**) Representative immunofluorescent confocal micrographs, demonstrating Cx43 (green) localization in control iPSCs (SOX2, iPSCs: purple) and after directed differentiation toward the three germ lineages (Nestin, ectoderm; Brachyury, mesoderm; SOX17, endoderm: purple) and nuclei (Hoechst, nuclei: blue). Cx43 forms large puncta (white arrows) at the cell surface indicative of gap junction plaques. Scale bars = 50 µm. (**B**) Representative Western blots and (**C**) densitometric analysis of total Cx43 protein expression in PAX6-positive ectoderm, Brachyury-positive mesoderm, and SOX17-positive endoderm cells. * *p* < 0.05 compared to undifferentiated iPSCs. Data represent the standard error of the mean of 3–4 independent experiments.

In line with the broad expression of Cx43 throughout the developing embryo, we found comparable Cx43 transcript expression across control iPSCs and all three embryonic germ lineages (Figure 1). Due to the widely reported role of Cx43 in stem cell differentiation [12,20,22,27,29], along with our findings that Cx43 transcripts are similarly expressed during iPSC lineage commitment, we focused on Cx43 protein expression and function across cells of the three germ lineages. Confocal microscopy and Western blot analyses of lineage-specific proteins demonstrate the successful differentiation of iPSCs to ectoderm (PAX6 or Nestin), endoderm (SOX17), and mesoderm (Brachyury) (Figure 2). Additionally, immunofluorescence confocal microscopy revealed Cx43 localized as small puncta, indicative of gap junction plaques, at the cellular interfaces of iPSCs and of each embryonic germ lineage (Figure 2A). Total Cx43 protein was not significantly different across undifferentiated iPSCs, ectodermal, or endodermal cells. However, in contrast to our qPCR results, iPSC differentiation toward mesoderm cells was accompanied by a significant increase in Cx43 protein compared to undifferentiated iPSCs (Figure 2B,C).

### 3.3. Human iPSCs Tolerate GJA1 CRISPR-Cas9 Editing

**Figure 3:**
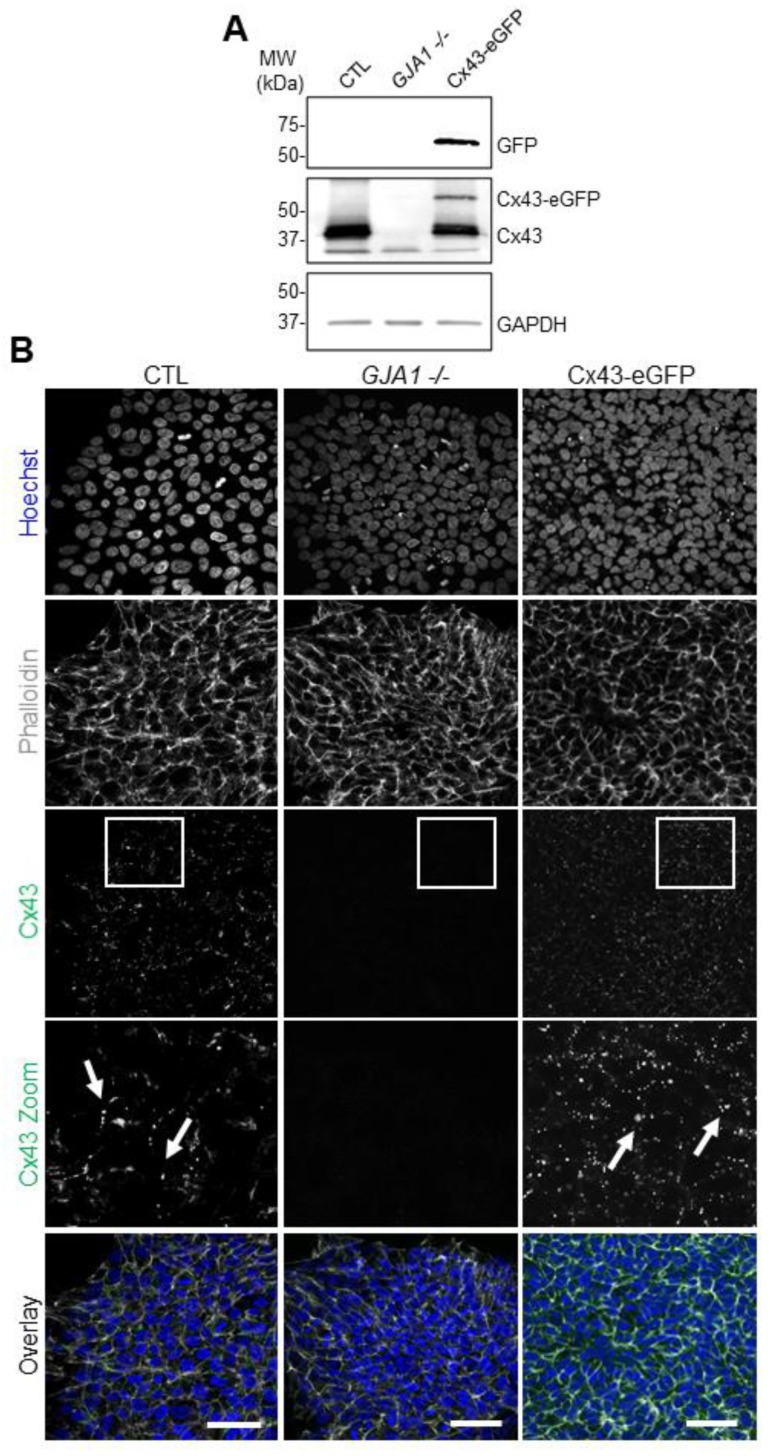
CRISPR-Cas9 manipulation of Cx43 (*GJA1*) in human iPSCs. (**A**) Representative Western blots of green fluorescence protein (GFP), Cx43, and GAPDH in control, Cx43 knockout (*GJA1-/-*), and Cx43eGFP iPSCs. (**B**) Immunofluorescence confocal microscopy of Cx43 (green) in control, *GJA1-/-*, and Cx43-eGFP iPSCs. Actin (phalloidin, grey); nuclei (Hoechst, blue). Cx43 forms large puncta (white arrows) at the cell surface indicative of gap junction plaques. Scale bars = 50 µm.

Previous studies demonstrated that Cx43 was dispensable for human iPSC survival and pluripotency gene expression [20]. However, due to the wide expression profile of Cx43 throughout early development, we sought to determine whether this protein plays a role in early cell fate specification. In addition to our previously published *GJA1-/-* (Cx43 knockout) iPSC line [20,28], we utilized Cx43-eGFP reporter iPSCs generated by the Allen Institute for Cell Science. This reporter line harbors a heterozygous insertion of the Cx43-eGFP construct at the endogenous *GJA1* allele, resulting in iPSCs with one wildtype *GJA1* allele and one genetically altered allele expressing Cx43-eGFP (Figure 3A). Importantly, the Cx43-eGFP iPSCs do not overexpress the reporter construct as the inserted Cx43-eGFP construct remains under control of the endogenous Cx43 promoter. Western blotting revealed bands of appropriate size for Cx43 (~43 kDa) in control iPSCs, which is absent in *GJA1-/-* iPSCs (Figure 3A). On the other hand, the Cx43-eGFP reporter cell line exhibits two distinct protein species corresponding to the endogenous Cx43 allele (~43 kDa) and the Cx43-eGFP knock-in allele (~60 kDa) (Figure 3A). Immunofluorescent confocal microscopy confirmed Cx43 protein expression at the cell surface in control human iPSCs, where Cx43 forms large puncta at opposing cell membranes (Figure 3B). As expected, punctate staining patterns are absent in *GJA1-/-* iPSCs (Figure 3B). Our Cx43-eGFP reporter iPSCs display comparable Cx43 expression and localization to control iPSCs, exhibiting large plasma membrane puncta (Figure 3B). Furthermore, we noted no observable difference in cellular morphology of the edited iPSCs compared to control (Figure 3B). Taken together, these three human iPSC lines (control, *GJA1-/-*, and Cx43-eGFP) enable a comprehensive evaluation of Cx43 during iPSC lineage commitment and differentiation.

### 3.4. Cx43-eGFP iPSCs Differentiate into All Three Germ Lineages

**Figure 4:**
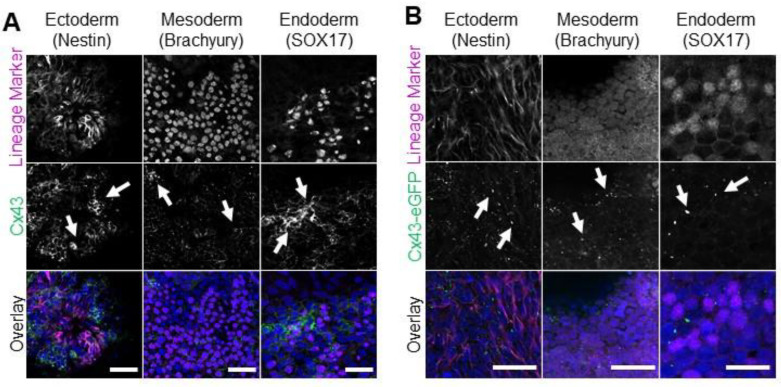
Control and Cx43-eGFP iPSCs spontaneously differentiate into cells from all three germ lineages. Representative immunofluorescence confocal micrographs demonstrating Cx43 or Cx43-eGFP (green) localization in control (**A**) or Cx43-eGFP reporter (**B**) iPSCs that underwent spontaneous differentiation into cells of each germ lineage: ectoderm (Nestin, purple), mesoderm (Brachyury, purple), or endoderm (SOX17, purple). Nuclei (Hoechst, blue). Cx43 forms large puncta (white arrows) at the cell surface indicative of gap junction plaques. Scale bars = 50 µm.

While the Cx43-eGFP human iPSC line exhibits comparable Cx43 expression levels and subcellular localization to control cells, it remained important to confirm that these genetically engineered iPSCs retained the ability to differentiate to the three embryonic germ layers. To that end, we performed 2-dimensional spontaneous monolayer iPSC differentiation to compare the inherent differentiation potential of the control iPSCs (Figure 4A) and Cx43-eGFP iPSCs (Figure 4B). Unlike directed differentiation, spontaneous differentiation does not utilize exogenous signals to drive cells toward specific lineages but instead relies on intrinsic cellular communication to confer fate decisions. Like control iPSCs, spontaneously differentiated Cx43-eGFP iPSCs successfully produced cells of each embryonic germ lineage, as identified by Nestin (ectoderm), Brachyury (mesoderm), and SOX17 (endoderm) expression (Figure 4A,B). Additionally, both control and Cx43-eGFP-differentiated cells expressed Cx43 across cells of each germ layer and formed the typical puncta indicative of gap junction plaques (Figure 4A,B).

### 3.5. Cx43 Is Dispensable during Lineage Specification

**Figure 5:**
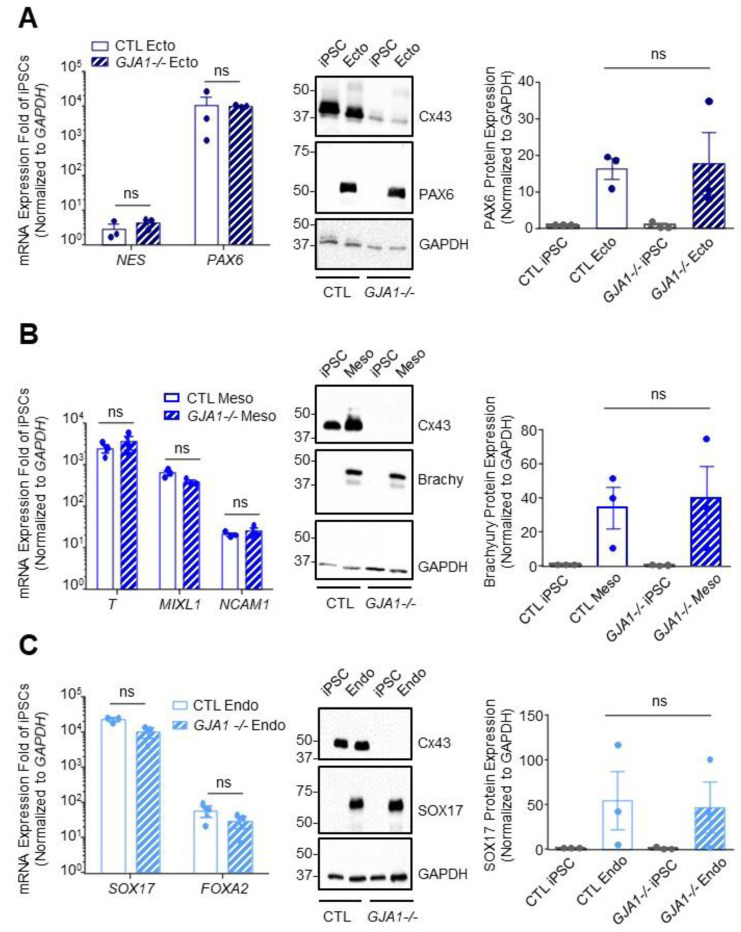
Cx43 knockout iPSCs successfully differentiate to cells of each germ lineage under directed differentiation conditions. Quantitative RT-PCR (qPCR) screen, representative Western blots, and densitometry of differentiated control and Cx43 knockout (*GJA1-/-*) iPSCs. (**A**) Ectoderm formation indicated by presence of *NES* and *PAX6* transcripts and expression of PAX6 protein. (**B**) Mesoderm evaluated by identifying transcripts for *T, MILX1*, and *NCAM1*, and expression of Brachyury protein. (**C**) Endoderm formation confirmed by *FOXA2* and *SOX17* transcripts and expression of SOX17 protein. Data represent the standard error of the mean of 3–4 independent experiments. ns: not significant compared to control cells.

**Figure 6:**
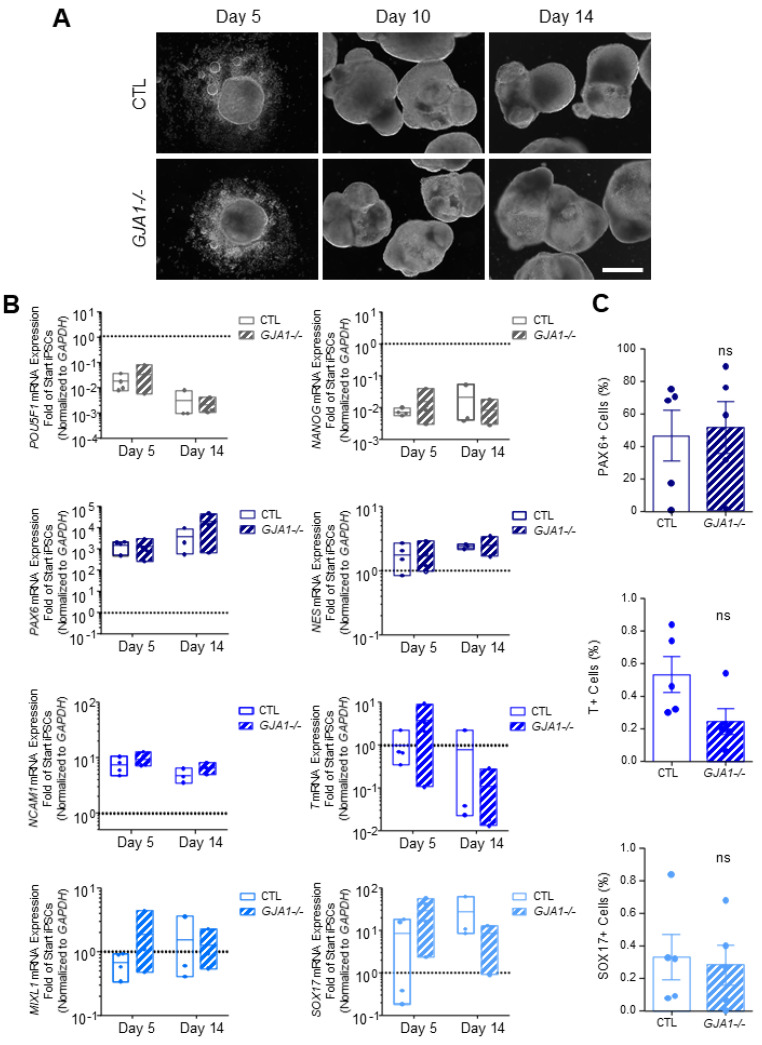
Spontaneously differentiated Cx43 knockout iPSCs form cells of all three germ lineages. (**A**) Phase contrast images of control and *GJA1-/-* embryoid bodies, scale bar 500 µm. (**B**) qPCR and (**C**) flow cytometric analyses demonstrate comparable spontaneous differentiation capacities of germ lineage populations by control and *GJA1-/-* embryoid bodies (pluripotency, grey; ectoderm, dark blue; mesoderm, blue; endoderm, light blue). ns: not significant compared to control cells.

Previous reports using pharmacological gap junction blockers or Cx43 siRNA knockdown suggest that Cx43 influences human and mouse PSC germ lineage specification [22,27]. To determine whether our *GJA1-/-* iPSCs exhibit similar germ lineage biases, we directed both control and *GJA1-/-* iPSCs to form ectoderm, endoderm, or mesoderm using a commercially available kit (Figure 5). In contrast to previous studies, we found no significant difference in ectoderm (PAX6; *PAX6*, *NES*), mesoderm (Brachyury; *T*, *MIXL1*, *NCAM1*), or endoderm (SOX17; *SOX17*, *FOXA2*) cells differentiated from either control or *GJA1-/-* iPSCs (Figure 5). In addition to directed differentiation, we compared the inherent differentiation preferences of control and *GJA1-/-* iPSCs using a 3-dimensional embryoid body (EB) model of spontaneous differentiation (Figure 6). Control and *GJA1-/-* iPSCs both successfully self-aggregated and formed EBs of comparable size when cultured in non-adherent cell culture dishes (Figure 6A). After 14 days of spontaneous differentiation, qPCR and flow cytometry revealed no significant difference in germ lineage-specific marker expression in EBs formed using control or *GJA1-/-* iPSCs (Figure 6B,C). Therefore, as *GJA1-/-* iPSCs successfully differentiated into all three germ lineages under both directed and spontaneous conditions, we conclude that Cx43 does not appear to skew human iPSC germ lineage specification. Inclusion of additional trophectoderm-containing models, such as gastruloids or blastoids, would allow future studies to further investigate the influence of Cxs on early cell signaling events regulating subsequent organization and patterning.

### 3.6. Other Connexin Isoforms Do Not Compensate for the Loss of Cx43 during Lineage Specification

**Figure 7:**
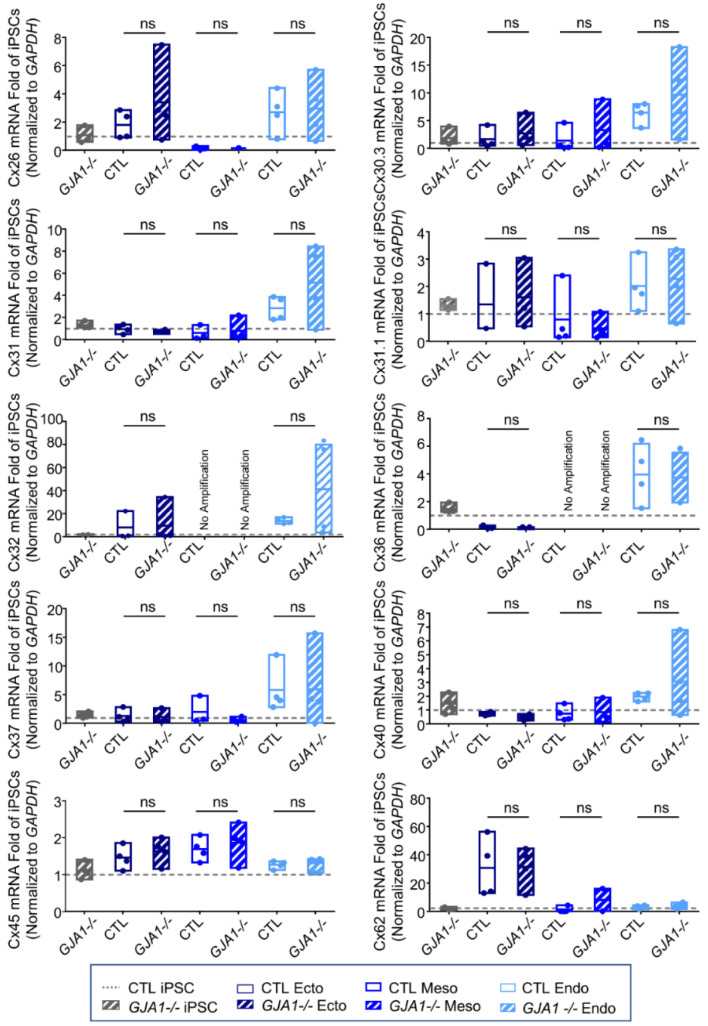
Other connexin isoforms do not compensate for the loss of Cx43 during iPSC lineage commitment. Quantitative RT-PCR (qPCR) screen of mRNA transcripts encoding 10 of the 21 human connexin isoforms in ectoderm, mesoderm, or endoderm directed differentiated from control and *GJA1-/-* iPSCs. Data represent the standard error of the mean of 4 independent experiments. ns: not significant compared to undifferentiated control iPSCs.

**Figure 8:**
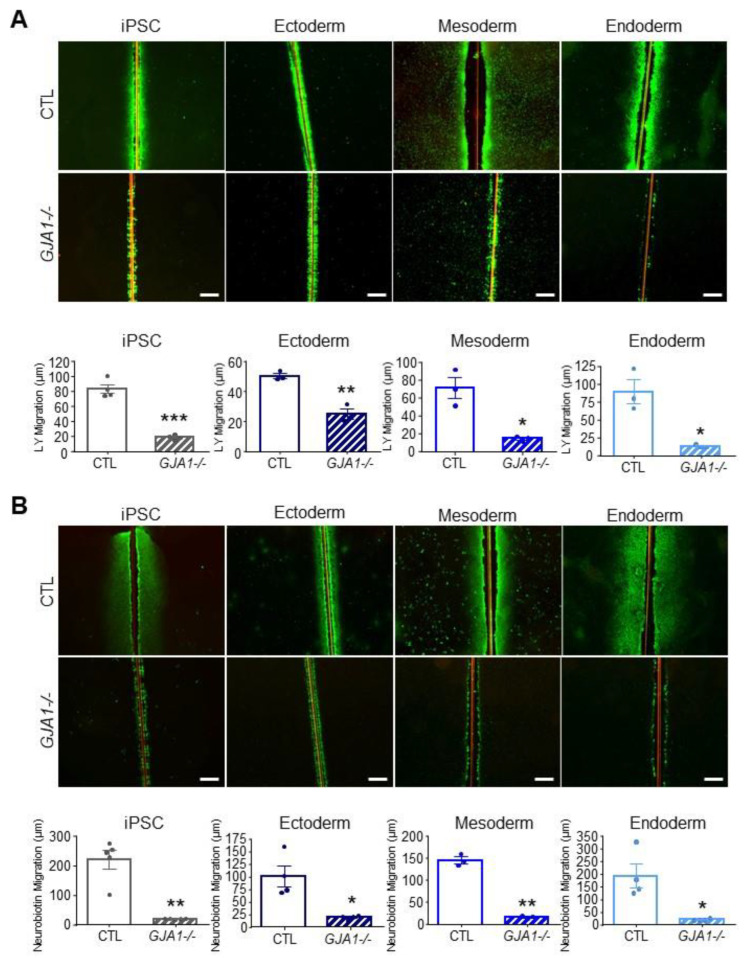
Gap junction coupling is lost in Cx43 knockout iPSCs and cells of each germ lineage. Scrape loading dye transfer assay of control and *GJA1-/-* iPSCs, ectoderm, mesoderm, and endoderm cells. (**A**) Representative fluorescence images of gap junction permeable dye Lucifer yellow (LY) or (**B**) neurobiotin. Scale bars = 200 µm. Data represent the standard error of the mean of 3–5 independent experiments. * *p* < 0.05; ** *p* < 0.01, *** *p* < 0.001 compared to control cells.

As described above, many connexin isoforms are expressed in human iPSCs and several are dynamically regulated during iPSC fate specification. Given that iPSCs, ectoderm, mesoderm, and endoderm lineages all tolerate the loss of Cx43 without apparent detriment (Figure 6), we investigated whether other Cx isoforms are upregulated to compensate for *GJA1* ablation. We found no significant difference in Cx transcript expression between control and *GJA1-/-* cells in any of the lineages investigated (Figure 7). Using gap junction-permeable dyes, Lucifer yellow (Figure 8A), and neurobiotin (Figure 8B), we performed scrape loading dye transfer assays to assess gap junction function in control and *GJA1-/-* iPSCs as well as the three germ layers. Control iPSCs exhibited an average Lucifer yellow dye transfer distance of 83.33 ± 6.14 µm and 221.1 ± 30.80 µm for the smaller neurobiotin tracer. In control ectoderm, we observed dye transfer distances of 50.47 ± 1.77 µm for Lucifer yellow and 100.90 ± 21.02 µm for neurobiotin. Meanwhile, control mesoderm cells displayed average dye transfer distances of 70.95 ± 11.59 µm for Lucifer yellow and 145.70 ± 7.63 µm for neurobiotin, and, finally, control endoderm-differentiated cells displayed 89.03 ± 16.64 µm Lucifer yellow transfer and 193.40 ± 46.31 µm for neurobiotin. In contrast to control cells, *GJA1-/-* iPSCs cells exhibited significantly reduced dye transfer in undifferentiated iPSCs, and after differentiation into each germ lineage relative to control counterparts. Together, these qPCR and dye transfer analyses suggest that *GJA1-/-* iPSCs do not upregulate other Cx isoforms to compensate for the loss of Cx43, even when directed to differentiate toward the three embryonic germ lineages.

## 4. Discussion

**Figure 9:**
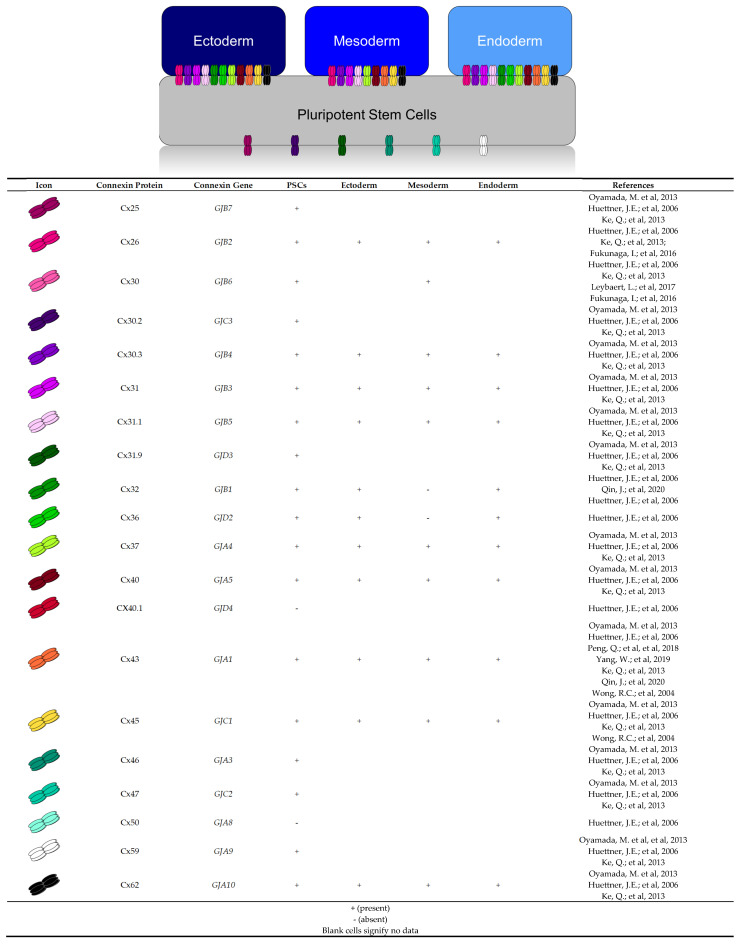
Human connexin expression profiles described in literature and this study. Human plu-ripotent stem cells express Cx40.1 [21] and Cx50 [21]. Cx23 expression in pluripotent stem cells and the three germ layers has not yet been characterized. Our study shows control iPSCs and the three germ layers express mRNA transcripts for Cx26 [21,38,51], Cx30.3 [7,21,38], Cx31 [7,21,38], Cx31.1 [7,21,38], Cx37 [7,21,38], Cx40 [7,21,38], Cx43 [7,21,22,27,38,43,44,45], Cx45 [7,21,38,45], and Cx62 [7,21,38]. Significant increases were viewed for mRNA transcripts of Cx62 in ectoderm; Cx45 in ectoderm and mesoderm; and for Cx30.3, Cx31, Cx32, Cx36, Cx37, and Cx40 in endo-derm. Transcripts for Cx32 and Cx36 were not detected in mesoderm.

Cell fate decisions are regulated, in part, by connexin-mediated gap junctional intercellular communication [20,29,39]. In this study, we examined the gene expression profiles for 11 of the 21 reported human Cx isoforms [39,40,41,42] in control human iPSCs and after differentiation toward the three embryonic germ layers. Our transcript analysis revealed several significant changes for select Cxs in ectoderm, mesoderm, or endoderm specification. Previous work in human iPSCs revealed gene expression of Cx25, Cx26, Cx30, Cx30.2, Cx30.3, Cx31, Cx31.1, Cx31.9, Cx32 [21,43], Cx36, Cx37, Cx40, Cx43, Cx45, Cx46, Cx47, Cx59, and Cx62 (summarized in Figure 9) [7,38]. On the other hand, only Cx43, and Cx45 have been identified at the protein level in human ESCs [21,44,45]. Our qPCR screen revealed expression of Cx26, Cx30.3, Cx31, Cx31.1, Cx32, Cx36, Cx37, Cx40, Cx43, Cx45, and Cx62 in control human iPSCs (Figure 1). Given the Cx expression profile revealed by ourselves and others, it remains possible that several different connexin isoforms work together to coordinate cell–cell communication in human iPSCs.

Like previous reports, we find that Cx43 is expressed in undifferentiated iPSCs and can be readily detected at the protein level in cells from all three germ lineages [22,27]. In iPSCs and the three germ layers, Cx43 protein localized to the cell surface, forming large gap junction plaques (Figure 2). Despite continued expression throughout differentiation, we find that Cx43 is dispensable for directed germ layer formation. This contrasts with previous studies which report that Cx43 is upregulated during endoderm differentiation and downregulated in ectoderm [22,27]. Indeed, the role of Cx43 in germ lineage specification includes several contrasting reports. Peng et al. 2019 demonstrated that shRNA knockdown of Cx43 has no impact on endoderm formation, as the knockdown cells were able to express definitive endoderm markers SOX17, FOXA2, and CXCR4 [22]. On the other hand, Yang et al. 2019 demonstrate that siRNA-mediated knockdown impedes definitive endoderm formation from human ESCs. Using CRISPR-Cas9 gene ablation, we find that Cx43 knockout iPSCs readily differentiate into all three germ lineages, with no significant difference from wildtype control cells. These discrepancies between our study and the previously published reports might result from our use of CRISPR-Cas9 gene editing rather than RNA interference knockdown methods. However, if that is the case, one would postulate that our knockout cells would exhibit a greater effect compared to previous knockdown studies. Another possibility is that other connexin isoforms could be upregulated to compensate for the loss of Cx43. As reviewed in Figure 9, few studies have investigated the presence of Cx proteins in iPSCs, and thereby the description of established GJIC in iPSCs remains limited. Gap junction coupling is commonly evaluated using small dyes and molecules, such as Lucifer yellow and neurobiotin [28,46]. Passage of these dyes and molecules is largely governed by channel composition, as it determines pore size, channel shape, and voltage gating. As such, various tracer dyes have been reported to be more permissible to Cx43 channels than Cx40 or Cx45 [47,48]. To broaden our investigation, we chose to use tracer dyes, Lucifer yellow and neurobiotin, as these molecules differ in their molecular weight and charge. Our screen of 10 other connexin isoforms, combined with our dye transfer experiments indicate that there is no GJIC compensation happening in our system. Future studies could make use of electrophysiological methods as a more definitive measure of gap junction coupling in Cx43 knockout iPSCs. Furthermore, future studies will determine whether the expression of any of the other Cxs not examined here are altered in our Cx43 CRISPR-ablated iPSCs. Finally, there is a growing body of work surrounding internally transcribed Cx43 isoforms [49]. Our CRISPR gRNAs target the first two thirds of the *GJA1* transcript, leaving the possibility of an internally transcribed Cx43 isoform which may continue to perform non-junctional functions within the cell.

While Cx43 has been well studied, there remains much to learn about the other 20 human Cx isoforms, especially given the link of certain Cxs to developmental processes and disease. For example, Cx40 mutations are associated with atrial fibrillation [50] and loss of functional Cx45 channels results in abnormal cardiomyocyte contraction and improper cardiac development [18]. Cx26 mutations are responsible for nearly half of all hereditary deafness cases [51], highlighting the importance of gap junctions for tissue function. To date, limited roles for connexins in stem cell fate decisions have been described. Overexpression of Cx43 in human ESCs positively mediates cardiac differentiation, while mesenchymal stem cells deficient in Cx43 exhibit decreased osteoblast differentiation potential [52,53]. Conversely, expression of Cx32 markedly decreases in the early stages of adipose-derived stem cell differentiation [54]. Interestingly, overexpression of Cx32 and knockdown of Cx43 promotes hepatocyte differentiation, highlighting the dynamic relationship between GJIC and cell fate specification [26]. Therefore, it is apparent that Cxs are important for the maintenance and downstream specification of somatic stem cell populations. Given that many Cxs isoforms are associated with human disease, understanding how they work at the cellular level will provide necessary insights into disease pathology but also reveal how they contribute to cellular differentiation and tissue patterning during development.

Implantation of the human embryo is followed by dramatic spatial rearrangements, such as those viewed during gastrulation. Large spatial rearrangement of post-implantation mouse embryos has been shown to be driven by positional cues passed between the embryonic epiblast and the extraembryonic trophectoderm [55,56]. Introducing newer, more sophisticated model systems into future studies would give a more accurate investigation of signaling processes occurring in vivo. The addition of blastoid and embryoid models would complement this study, through the inclusion of influential trophectoderm specific Cx-mediated signals [57,58]. Furthermore, models, such as gastruloids, would add spatioaxial signals possibly lacking in our current embryoid body models [59].

Several reports dispute the role of Cxs in the establishment, survival, and maintenance of human PSCs [7,20,22,29,38,60,61,62]. For example, dye transfer assays demonstrate the reestablishment of GJIC during iPSC reprogramming [62], and siRNA knockdown of either Cx43 or Cx45 has been shown to negatively impact iPSC reprogramming efficiency [38,60]. Furthermore, ectopic expression of Cx43 and Cx45 enhances iPSC reprogramming efficiency [29,38,60,63,64]. However, while broad-spectrum GJIC pharmacological inhibition via carbenoxolone kills human iPSCs, it appears that this occurs independently from Cx43 as our *GJA1-/-* iPSCs exhibit comparable morphology and survival in culture relative to control iPSCs (Figure 3) [20]. Comprehensive evaluation of how different Cx isoforms contribute to human PSC survival and potency will help resolve these discrepancies. Additional studies using precise genetic ablation of various connexin isoforms, rather than broad pharmacological blockade, will clarify the role played by Cxs in regulating the self-renewal and differentiation capacity of human pluripotent stem cells.

## 5. Conclusions

In conclusion, we present the differential transcript expression for 11 of the 21 Cx isoforms in iPSCs and the three germ layers. Despite significant upregulation of several Cx transcripts in various germ lineages, along with significant upregulation of Cx43 protein in mesoderm, we conclude that Cx43-mediated GJIC is dispensable during lineage specification of human iPSCs.

## Figures and Tables

**Table 1:** Primary and secondary antibodies and dyes used in immunofluorescence.

Target Protein	Host Species or Dye	Antibody Type & Fluorophore	Dilution	Cat# & Vendor
Actin	Dye	Phalloidin (AF555)	1:500	A34055, ThermoFisher
Brachyury	Rabbit	Primary	1:1000	EPR18113, Abcam (Cambridge, UK)
Brachyury	Goat	Primary	1:500	IC2085G, R&D Systems (Minneapolis, MN, USA)
Connexin43	Rabbit	Primary	1:1000	C6219, Sigma-Aldrich
Nestin	Mouse	Primary	1:500	14-9843-82, ThermoFisher
Nuclei	Dye	Hoechst 33342	1:1000	H3570, Fisher Scientific
SOX17	Goat	Primary	1:500	AF1924, R&D Systems
SOX2	Mouse	Primary	1:200	AF2018, R&D Systems
Goat	Donkey	Secondary (AF555)	1:500	AS32816, ThermoFisher
Goat	Donkey	Secondary (AF647)	1:500	A21082, ThermoFisher
Mouse	Donkey	Secondary (AF488)	1:500	A31572, ThermoFisher
Mouse	Donkey	Secondary (AF555)	1:500	A31570, ThermoFisher
Mouse	Goat	Secondary (AF647)	1:500	A32728, ThermoFisher
Rabbit	Donkey	Secondary (AF488)	1:500	A21206, ThermoFisher
Rabbit	Donkey	Secondary (AF555)	1:500	A31572, ThermoFisher
Rabbit	Donkey	Secondary (AF647)	1:500	A31573, ThermoFisher

**Table 2:** Primary and secondary antibodies used in western blotting.

Target	Host Species	Antibody & Fluorophore	Dilution	Cat# & Vendor
Brachyury (T)	Rabbit	Primary	1:1000	ab209665, Abcam
Connexin43	Rabbit	Primary	1:2000	C6219, Sigma-Aldrich
GAPDH	Mouse	Primary	1:5000	MAB374, Sigma-Aldrich
PAX6	Rabbit	Primary	1:1000	ab195045, Abcam
SOX17	Goat	Primary	1:1000	AF1924, R&D Systems
Goat	Donkey	Secondary (HRP)	1:1000	Mearow Lab
Mouse	Goat	Secondary (HRP)	1:1000	31430, ThermoFisher
Rabbit	Goat	Secondary (HRP)	1:1000	31460, ThermoFisher

**Table 3:** Primer sets used in qPCR analyses.

Target	Forward (5’-3’)	Reverse (5’-3’)	Product Size (bp)
*FOXA2*	Hs.PT.58.22972176		
*GAPDH*	TGCTTTTAACTCTGGTAAAG	CACTTGATTTTGGAGGGATC	198
*GJA1 (Cx43)*	GGTCTGAGTGCCTGAACTTGCCT	AGCCACACCTTCCCTCCAGCA	184
*GJA10 (Cx62)*	AGGCAACTTGAACTAGACCCTT	GCCGTAGTTGTACCTAGCCA	197
*GJA4 (Cx37)*	TCAGCACACCCACCCTGGTCT	GGATGCGCAGGCGACCATCTT	189
*GJA5 (Cx40)*	CCCAGTATACGAAGCCTTTC	TTTGGTATGCTGCTGGTATG	136
*GJB1 (Cx32)*	AGGTCCACATCTCAGGGACA	AAGACGGTTTTCTCGGTGGG	199
*GJB2 (Cx26)*	CCCGACGCAGAGCAAAC	CAGGGTGCAGACAAAGT	200
*GJB3 (Cx31)*	CACTCTCTGGCATGGCTTCA	GTAGGTCGGGCAATGTAGCA	96
*GJB4 (Cx30.3)*	TGTGGTGGACGTACTTGCTG	GCGGGGCATGTCATAATCCT	101
*GJB5 (Cx31.1)*	AAACAAGACGACCTCCTTTC	CCCTCACAAGATGGTTTTCT	111
*GJC1 (Cx45)*	TACACCGAACTGTCCAATGC	TCCCATCCCCTGATTTGCTA	271
*GJD2 (Cx36)*	AGCAGCACTCCACTATGATCG	GTAGAGTAGCGGCGTTCTCG	286
*MIXL1*	GCTTTCAGTTACCCTCCCAGA- TAAC	GCACAGGAAGTACATAACAA- GTGC	270
*NANOG*	TGCTGAGATGCCTCACACGGA	TGACCGGGACCTTGTCTTCCTT	155
*NCAM1*	GCCTGAAGCCCGAAACAAC	CACTGGGTTCCCCTTGGA	117
*NES*	CTGCGGGCTACTGAAAAGTT	TCCAGGAGGGTCCTGTACG	161
*PAX6*	CAGCTCGGTGGTGTCTTTG	CCGTTGGACACGTTTTGATTG	167
*POU4F1 (OCT4)*	TGGGCTCGAGAAGGATGTG	GCATAGTCGCTGCTTGATCG	78
*SOX17*	GAGCCAAGGGCGAGTCCCGTA	CCTTCCACGACTTGCCCAGCAT	141
*T (Brachyury)*	Hs.PT.58.1243965		

**Table 4:** Antibodies used in flow cytometry.

Target	Host Species	Fluorophore	Dilution	Cat# & Vendor
Brachyury (T)	Goat	AF488	1:50	IC2085G, R&D Systems
PAX6	Mouse	PerCP-Cy5.5	1:200	562388, BD Biosciences, Franklin Lakes, NJ, USA
SOX17	Goat	APC	1:200	IC1924A, R&D Systems

**Table 5:** Expression patterns of 11 Cx isoforms in control human iPSCs and three germ layers.

Connexin Protein	Connexin Gene (Human)	iPSCs	Ectoderm	Mesoderm	Endoderm
Cx26	*GJB2*	+	+	+	+
Cx30.3	*GJB4*	+	+	+	+ + +
Cx31	*GJB3*	+	+	+	+ + +
Cx31.1	*GJB5*	+	+	+	+
Cx32	*GJB1*	+	+	nd	+ + +
Cx36	*GJD2*	+	+	nd	+ + +
Cx37	*GJA4*	+	+	+	+ + +
Cx40	*GJA5*	+	+	+	+ + +
Cx43	*GJA1*	+	+	+	+
Cx45	*GJC1*	+	+ + +	+ + +	+
Cx62	*GJA10*	+	+ + +	+	+

Expression relative to iPSCs: + (present–no statistical difference from iPSCs); + + + (present–statistically elevated from iPSCs), nd (absent–qPCR nondetect).

## Data Availability

The raw data supporting the conclusions of this article will be made available by the authors, without undue reservation.

## References

[1] Wörsdörfer P., Maxeiner S., Markopoulos C., Kirfel G., Wulf V., Auth T., Urschel S., von Maltzahn J., Willecke K. (2008). Connexin Expression and Functional Analysis of Gap Junctional Communication in Mouse Embryonic Stem Cells. Stem Cells.

[2] Esseltine J.L., Laird D.W. (2016). Next-Generation Connexin and Pannexin Cell Biology. Trends Cell Biol..

[3] Laird D.W. (2006). Life Cycle of Connexins in Health and Disease. Biochem. J..

[4] Bevans C.G., Kordel M., Rhee S.K., Harris A. (1998). Isoform Composition of Connexin Channels Determines Selectivity among Second Messengers and Uncharged Molecules. J. Biol. Chem..

[5] Harris A.L., Contreras J.E. (2014). Motifs in the Permeation Pathway of Connexin Channels Mediate Voltage and Ca^2+^ Sensing. Front. Physiol..

[6] Vitorín J.F.E., Pontifex T.K., Burt J.M. (2016). Determinants of Cx43 Channel Gating and Permeation: The Amino Terminus. Biophys. J..

[7] Oyamada M., Takebe K., Endo A., Hara S., Oyamada Y. (2013). Connexin Expression and Gap-Junctional Intercellular Communication in ES Cells and iPS Cells. Front. Pharmacol..

[8] Laird D.W., Naus C.C., Lampe P.D. (2017). SnapShot: Connexins and Disease. Cell.

[9] Becker D.L., Leclerc-David C., Warner A. (1992). The Relationship of Gap Junctions and Compaction in the Preimplantation Mouse Embryo. Development.

[10] Wei C.-J., Francis R., Xu X., Lo C.W. (2005). Connexin43 Associated with an N-Cadherin-Containing Multiprotein Complex Is Required for Gap Junction Formation in NIH3T3 Cells. J. Biol. Chem..

[11] Lee S., Gilula N., Warner A. (1987). Gap Junctional Communication and Compaction during Preimplantation Stages of Mouse Development. Cell.

[12] Wong R.C.B., Pera M.F., Pébay A. (2008). Role of Gap Junctions in Embryonic and Somatic Stem Cells. Stem Cell Rev. Rep..

[13] Wörsdörfer P., Wagner N., Ergün S. (2018). The Role of Connexins During Early Embryonic Development: Pluripotent Stem Cells, Gene Editing, and Artificial Embryonic Tissues as Tools to Close the Knowledge Gap. Histochem. Cell Biol..

[14] Bloor D.J., Wilson Y., Kibschull M., Traub O., Leese H.J., Winterhager E., Kimber S.J. (2004). Expression of Connexins in Human Preimplantation Embryos in Vitro. Reprod. Biol. Endocrinol..

[15] Hardy K., Warner A., Winston R.M., Becker D.L. (1996). Expression of Intercellular Junctions during Preimplantation Development of the Human Embryo. Mol. Hum. Reprod..

[16] Gabriel H.-D., Jung D., Bützler C., Temme A., Traub O., Winterhager E., Willecke K. (1998). Transplacental Uptake of Glucose Is Decreased in Embryonic Lethal Connexin26-Deficient Mice. J. Cell Biol..

[17] Reaume A., De Sousa P., Kulkarni S., Langille B., Zhu D., Davies T., Juneja S., Kidder G., Rossant J. (1995). Cardiac Malformation in Neonatal Mice Lacking Connexin43. Science.

[18] Nishii K., Kumai M., Egashira K., Miwa T., Hashizume K., Miyano Y., Shibata Y. (2003). Mice Lacking Connexin45 Conditionally in Cardiac Myocytes Display Embryonic Lethality Similar to That of Germline Knockout Mice Without Endocardial Cushion Defect. Cell Commun. Adhes..

[19] Houghton F.D. (2005). Role of Gap Junctions during Early Embryo Development. Reproduction.

[20] Esseltine J.L., Brooks C.R., Edwards N.A., Subasri M., Sampson J., Séguin C., Betts D.H., Laird D.W. (2019). Dynamic Regulation of Connexins in Stem Cell Pluripotency. Stem Cells.

[21] Huettner J.E., Lu A., Qu Y., Wu Y., Kim M., McDonald J.W. (2006). Gap Junctions and Connexon Hemichannels in Human Embryonic Stem Cells. Stem Cells.

[22] Peng Q., Yue C., Chen A.C.H., Lee K.C., Fong S.W., Yeung W.S.B., Lee Y.L. (2018). Connexin 43 Is Involved in Early Differentiation of Human Embryonic Stem Cells. Differentiation.

[23] Loh K.M., Ang L.T., Zhang J., Kumar V., Ang J., Auyeong J.Q., Lee K.L., Choo S.H., Lim C.Y., Nichane M. (2014). Efficient Endoderm Induction from Human Pluripotent Stem Cells by Logically Directing Signals Controlling Lineage Bifurcations. Cell Stem Cell.

[24] Gilbert S.F. (2000). Developmental Biology.

[25] Qin J., Chang M., Wang S., Liu Z., Zhu W., Wang Y., Yan F., Li J., Zhang B., Dou G. (2016). Connexin 32-Mediated Cell-Cell Communication Is Essential for Hepatic Differentiation from Human Embryonic Stem Cells. Sci. Rep..

[26] Pei H., Zhai C., Li H., Yan F., Qin J., Yuan H., Zhang R., Wang S., Zhang W., Chang M. (2017). Connexin 32 and Connexin 43 Are Involved in Lineage Restriction of Hepatic Progenitor Cells to Hepatocytes. Stem Cell Res. Ther..

[27] Yang W., Lampe P.D., Kensel-Hammes P., Hesson J., Ware C.B., Crisa L., Cirulli V. (2019). Connexin 43 Functions as a Positive Regulator of Stem Cell Differentiation into Definitive Endoderm and Pancreatic Progenitors. iScience.

[28] Shao Q., Esseltine J.L., Huang T., Novielli-Kuntz N., Ching J.E., Sampson J., Laird D.W. (2019). Connexin43 is Dispensable for Early Stage Human Mesenchymal Stem Cell Adipogenic Differentiation But Is Protective against Cell Senescence. Biomolecules.

[29] Esseltine J.L., Shao Q., Brooks C., Sampson J., Betts D.H., A Séguin C., Laird D.W. (2017). Connexin43 Mutant Patient-Derived Induced Pluripotent Stem Cells Exhibit Altered Differentiation Potential. J. Bone Miner. Res..

[30] Claassen D., Desler M.M., Rizzino A. (2009). ROCK Inhibition Enhances the Recovery and Growth of Cryopreserved Human Embryonic Stem Cells and Human Induced Pluripotent Stem Cells. Mol. Reprod. Dev..

[31] Mullen A.C., Wrana J.L. (2017). TGF-β Family Signaling in Embryonic and Somatic Stem-Cell Renewal and Differentiation. Cold Spring Harb. Perspect. Biol..

[32] Tabari M.G., Jorsaraei S.G.A., Ghasemzadeh-Hasankolaei M., Ahmadi A.A., Ghasemi M. (2019). Comparison of Germ Cell Gene Expressions in Spontaneous Monolayer versus Embryoid Body Differentiation of Mouse Embryonic Stem Cells toward Germ Cells. Int. J. Fertil. Steril..

[33] Dahlmann J., Kensah G., Kempf H., Skvorc D., Gawol A., Elliott D., Dräger G., Zweigerdt R., Martin U., Gruh I. (2013). The Use of Agarose Microwells for Scalable Embryoid Body Formation and Cardiac Differentiation of Human and Murine Pluripotent Stem Cells. Biomaterials.

[34] Friedrich J., Seidel C., Ebner R., Kunz-Schughart L.A. (2009). Spheroid-Based Drug Screen: Considerations and Practical Approach. Nat. Protoc..

[35] Laboratory C.S.H. (2007). Mowial-DABCO Stock Solution. Cold Spring Harb. Protoc..

[36] Schindelin J., Arganda-Carreras I., Frise E., Kaynig V., Longair M., Pietzsch T., Preibisch S., Rueden C., Saalfeld S., Schmid B. (2012). Fiji: An Open-Source Platform for Biological-Image Analysis. Nat. Methods.

[37] Bolte S., Cordelières F.P. (2006). A Guided Tour into Subcellular Colocalization Analysis in Light Microscopy. J. Microsc..

[38] Ke Q., Li L., Cai B., Liu C., Yang Y., Gao Y., Huang W., Yuan X., Wang T., Zhang Q. (2013). Connexin 43 Is Involved in the Generation of Human-Induced Pluripotent Stem Cells. Hum. Mol. Genet..

[39] Genet N., Bhatt N., Bourdieu A., Hirschi K.K. (2018). Multifaceted Roles of Connexin 43 in Stem Cell Niches. Curr. Stem Cell Rep..

[40] Goodenough D.A., Paul D.L. (2009). Gap Junctions. Cold Spring Harb. Perspect. Biol..

[41] Söhl G. (2004). Gap Junctions and the Connexin Protein Family. Cardiovasc. Res..

[42] Aasen T., Mesnil M., Naus C.C., Lampe P.D., Laird D.W. (2016). Gap Junctions and Cancer: Communicating for 50 Years. Nat. Rev. Cancer.

[43] Qin J., Chang M., Wang S., Liu Z., Zhu W., Wang Y., Yan F., Li J., Zhang B., Dou G. (2020). Author Correction: Connexin 32-Mediated Cell-Cell Communication Is Essential for Hepatic Differentiation from Human Embryonic Stem Cells. Sci. Rep..

[44] Wong R.C., Pébay A., Nguyen L.T., Koh K.L., Pera M.F. (2004). Presence of Functional Gap Junctions in Human Embryonic Stem Cells. Stem Cells.

[45] Carpenter M.K., Rosler E.S., Fisk G.J., Brandenberger R., Ares X., Miura T., Lucero M., Rao M.S. (2003). Properties of Four Human Embryonic Stem Cell Lines Maintained in a Feeder-Free Culture System. Dev. Dyn..

[46] Mills S.L., Massey S.C. (2000). A Series of Biotinylated Tracers Distinguishes Three Types of Gap Junction in Retina. J. Neurosci..

[47] Weber P.A., Chang H.-C., Spaeth K.E., Nitsche J.M., Nicholson B. (2004). The Permeability of Gap Junction Channels to Probes of Different Size Is Dependent on Connexin Composition and Permeant-Pore Affinities. Biophys. J..

[48] Elfgang C., Eckert R., Lichtenberg-Fraté H., Butterweck A., Traub O., A Klein R., Hülser D.F., Willecke K. (1995). Specific Permeability and Selective Formation of Gap Junction Channels in Connexin-Transfected HeLa Cells. J. Cell Biol..

[49] Smyth J., Shaw R.M. (2013). Autoregulation of Connexin43 Gap Junction Formation by Internally Translated Isoforms. Cell Rep..

[50] Leybaert L., Lampe P.D., Dhein S., Kwak B., Ferdinandy P., Beyer E., Laird D.W., Naus C.C., Green C.R., Schulz R. (2017). Connexins in Cardiovascular and Neurovascular Health and Disease: Pharmacological Implications. Pharmacol. Rev..

[51] Fukunaga I., Fujimoto A., Hatakeyama K., Aoki T., Nishikawa A., Noda T., Minowa O., Kurebayashi N., Ikeda K., Kamiya K. (2016). In Vitro Models of GJB2-Related Hearing Loss Recapitulate Ca2+ Transients via a Gap Junction Characteristic of Developing Cochlea. Stem Cell Rep..

[52] Moore J.C., Tsang S.-Y., Rushing S.N., Lin D., Tse H.F., Chan C.W., Li R.A. (2008). Functional Consequences of Overexpressing the Gap Junction Cx43 in the Cardiogenic Potential of Pluripotent Human Embryonic Stem Cells. Biochem. Biophys. Res. Commun..

[53] Lin F.-X., Zheng G.-Z., Chang B., Chen R.-C., Zhang Q.-H., Xie P., Xie D., Yu G.-Y., Hu Q.-X., Liu D.-Z. (2018). Connexin 43 Modulates Osteogenic Differentiation of Bone Marrow Stromal Cells through GSK-3beta/Beta-Catenin Signaling Pathways. Cell. Physiol. Biochem..

[54] Mannino G., Vicario N., Parenti R., Giuffrida R., Furno D.L. (2020). Connexin Expression Decreases during Adipogenic Differentiation of Human Adipose-Derived Mesenchymal Stem Cells. Mol. Biol. Rep..

[55] Christodoulou N., Weberling A., Strathdee D., Anderson K.I., Timpson P., Zernicka-Goetz M. (2019). Morphogenesis of Extra-Embryonic Tissues Directs the Remodelling of the Mouse Embryo at Implantation. Nat. Commun..

[56] Weberling A., Zernicka-Goetz M. (2021). Trophectoderm Mechanics Direct Epiblast Shape upon Embryo Implantation. Cell Rep..

[57] Niemann H., Seamark B. (2021). Blastoids: A New Model for Human Blastocyst Development. Signal Transduct. Target. Ther..

[58] Warmflash A., Sorre B., Etoc F., Siggia E.D., Brivanlou A.H. (2014). A Method to Recapitulate Early Embryonic Spatial Patterning in Human Embryonic Stem Cells. Nat. Methods.

[59] Moris N., Anlas K., Brink S.C.V.D., Alemany A., Schröder J., Ghimire S., Balayo T., van Oudenaarden A., Arias A.M. (2020). An in Vitro Model of Early Anteroposterior Organization during Human Development. Nature.

[60] Ke Q., Li L., Yao X., Lai X., Cai B., Chen H., Chen R., Zhai Z., Huang L., Li K. (2017). Enhanced Generation of Human Induced Pluripotent Stem Cells by Ectopic Expression of Connexin 45. Sci. Rep..

[61] Wong R.C.-B., Dottori M., Koh K.L., Nguyen L.T., Pera M.F., Pébay A. (2006). Gap Junctions Modulate Apoptosis and Colony Growth of Human Embryonic Stem Cells Maintained in a Serum-Free System. Biochem. Biophys. Res. Commun..

[62] Sharovskaya Y.Y., Philonenko E.S., Kiselev S.L., Lagarkova M.A. (2012). De Novo Reestablishment of Gap Junctional Intercellular Communications during Reprogramming to Pluripotency and Differentiation. Stem Cells Dev..

[63] Dbouk H.A., Mroue R.M., El-Sabban M.E., Talhouk R.S. (2009). Connexins: A Myriad of Functions Extending beyond Assembly of Gap Junction Channels. Cell Commun. Signal..

[64] Czyż J., Piwowarczyk K., Paw M., Luty M., Wróbel T., Catapano J., Madeja Z., Ryszawy D. (2017). Connexin-Dependent Intercellular Stress Signaling in Tissue Homeostasis and Tumor Development. Acta Biochim. Pol..

